# Preparation, Characterization and Antioxidant Activities of Kelp Phlorotannin Nanoparticles

**DOI:** 10.3390/molecules25194550

**Published:** 2020-10-05

**Authors:** Ying Bai, Yihan Sun, Yue Gu, Jie Zheng, Chenxu Yu, Hang Qi

**Affiliations:** 1 School of Food Science and Technology, Dalian Polytechnic University, National Engineering Research Center of Seafood, Liaoning Provincial Aquatic Products Deep Processing Technology Research Center, Dalian 116034, China; 18340837366@163.com (Y.B.); s18641423774@163.com (Y.S.); 713guyue@sina.com (Y.G.); 2 Liaoning Ocean and Fisheries Science Research Institute, Dalian 116023, China; zhengjiessd@163.com; 3 Department of Agricultural and Biosystems Engineering, Iowa State University, Ames, IA 50011, USA; chenxuyu@iastate.edu

**Keywords:** phlorotannin, encapsulation, characterization, stability, antioxidant activity

## Abstract

Phlorotannins are a group of major polyphenol secondary metabolites found only in brown algae and are known for their bioactivities and multiple health benefits. However, they can be oxidized due to external factors and their bioavailability is low due to their low water solubility. In this study, the potential of utilizing nanoencapsulation with polyvinylpyrrolidone (PVP) to improve various activities of phlorotannins was explored. Phlorotannins encapsulated by PVP nanoparticles (PPNPS) with different loading ratios were prepared for characterization. Then, the PPNPS were evaluated for in vitro controlled release of phlorotannin, toxicity and antioxidant activities at the ratio of phlorotannin to PVP 1:8. The results indicated that the PPNPS showed a slow and sustained kinetic release of phlorotannin in simulated gastrointestinal fluids, they were non-toxic to HaCaT keratinocytes and they could reduce the generation of endogenous reactive oxygen species (ROS). Therefore, PPNPS have the potential to be a useful platform for the utilization of phlorotannin in both pharmaceutical and cosmetics industries.

## 1. Introduction

In recent years, research on bioactive substances originated from algae has increased rapidly. These compounds not only can serve as preservatives and antioxidant protectants in food and cosmetics, but also show multiple health benefits [1]. Among these active compounds, polyphenolic phlorotannins, a group of complex molecular assemblages from the polymerization of phloroglucinol [2], represent a large class of well-characterized brown algae secondary metabolites [3]. They have been revealed to have a range of diverse biological functions and activities, such as antibacterial [4], anti-inflammatory [5], anti-oxidant [6], antidiabetic [7], anti-HIV [8] and anticancer [9,10,11] activities. However, phlorotannins are highly sensitive to external factors such as light, pH, heat and oxidative reagents, which limit their applications in food, cosmetic, nutraceutical and pharmaceutical industries [12]. In addition, low water solubility of phlorotannin leads to low absorption in the gastrointestinal tract, which affects their bioavailability [13,14].

Encapsulation is a common technique to wrap a core material with functional activity in a coating material to form a capsule. This technology can not only mask or retain flavor and increase solubility, but also protects the core material from degradation by environmental factors and controls the release of various biologically active compounds at the target site [15]. Solid dispersion technique is one of the encapsulation methods that has been applied to various drugs and natural bioactive substances [16]. To apply such a technique on phlorotannin, it is important to fully investigate the effects of encapsulation on the functional properties of phlorotannin. In the preparation of solid dispersion, a water-soluble carrier such as high-molecular-weight polyvinylpyrrolidone (PVP) would be a good choice as it has low toxicity, good physiological compatibility and excellent thermophysical properties which will facilitate its application in medicine, food and cosmetics [17]. PVP is an amorphous, polar polymer, well soluble in water and some organic solvents, and is able to form complexes with other substances. It is also a good stabilizer, which can prevent the aggregation of nanoparticles through the repulsive force formed by its hydrophobic carbon chains, which extend into the solvents and interact with each other [18]. These characteristics make PVP possess better wall material advantages in preparing nanoparticles compared other chemicals. The objectives of this study were two-fold: (1) to characterize physicochemical properties of phlorotannin encapsulated with PVP nanoparticles (PPNPS) and to evaluate the improvement over stability and the retention rate of active phlorotannins by PPNPS in the in vitro digestion process; and (2) to analyze the PPNPS cytotoxicity and antioxidant activities using a keratinocyte model to evaluate the potential of PPNPS as an anti-oxidative protectant, which potentially can be used in skincare products.

## 2. Results and Discussion

### 2.1. Characterization of PPNPS

**Figure 1:**
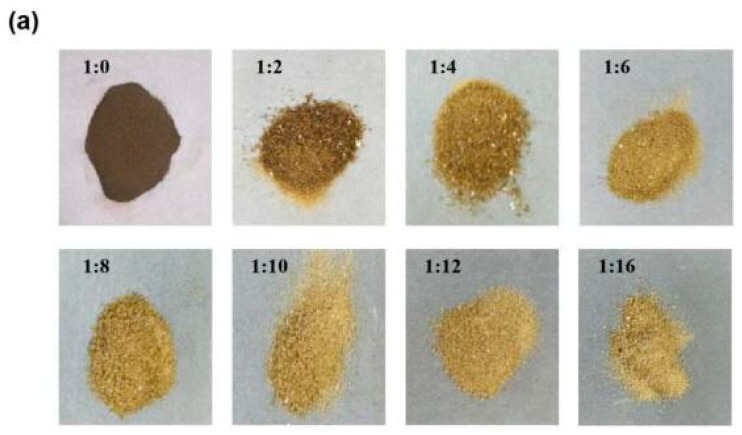

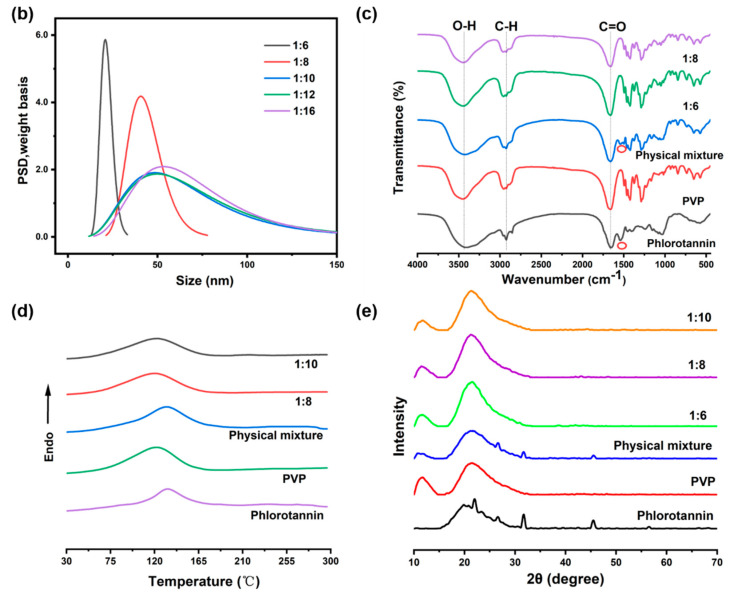
Characterization of PPNPS (polyvinylpyrrolidone nanoparticles): (**a**) Images of PPNPS with different loading ratios; (**b**) Particle size distribution (the concentration of PPNPS was 10 mg/mL in solution); (**c**) FTIR spectra (the samples were solid); (**d**) DSC (differential scanning calorimetry) thermograms (the samples were solid) and (**e**) X-ray diffraction patterns (the samples were solid).

In order to evaluate the stability of the PPNPS, the nanoparticles were prepared with different loading ratios of phlorotannin:PVP. As shown in Figure 1a, images of PPNPS samples with different loading indicated that as the PVP concentration increased, the color of the PPNPS became lighter. This suggested that more and more phlorotannins were embedded inside the PVP shell and fewer exposed phlorotannins remained on the surface of the PPNPS. These results were consistent with the embedding rate from 1:6 to 1:16 (phlorotannin: PVP, *w/w*) analyzed using high-performance liquid chromatography (HPLC, Shimadzu SPD 20A, Kyoto, Japan). The embedding rates were 89.56 ± 1.21%, 89.22 ± 0.81%, 88.56 ± 1.35%, 88.92 ± 1,15% and 89.47 ± 1.32%, respectively (1:6, 1:8, 1:10, 1:12, 1:16) (data not shown).

Particle size distribution (Figure 1b) of PPNPS with loading ratios of phlorotannin:PVP (*w/w*) of 1:6, 1:8, 1:10, 1:12, 1:16 (*w/w*) showed that as the PVP content increased, the average size of the PPNPS appeared to gradually increase, while the uniformity of the PPNPS declined and broader distribution was observed. It is reasonable that as the PVP concentration gradually increased, the shell became thicker. D_50_ values of 1:6, 1:8, 1:10, 1:12, 1:16 were 20.84 ± 0.94, 40.62 ± 2.55, 48.32 ± 2.39, 49.66 ± 2.71, 53.17 ± 2.47 nm, respectively. This suggested that encapsulation of PVP caused an expansion of the nanoparticles, which is a common phenomenon reported in the literature [19]. The FTIR measurement was performed to verify the encapsulation of phlorotannin inside the PVP shell [20]. As shown in Figure 1c, when the FTIR spectra of the PPNPS, phlorotannin and phlorotannin + PVP mixture were compared, it was clear that some of the peaks associated with specific functional groups in the phlorotannin molecules (e.g., 1546 cm^−1^) only appeared in the spectrum of the mixture, but not in that of the PPNPS. Apparently, the encapsulation of the phlorotannin was successful in the PPNPS (1:6 and 1:8) and only the PVP (the shell in the PPNPS) bands were observed in the representative FTIR spectrum of PPNPS. However, the FTIR spectrum of the physical mixture sample showed both phlorotannin and PVP characteristic peaks. These characteristic peaks in the physical mixture samples also supported the encapsulation of phlorotannin. The result was similar to Zhu et al.’s previous report that fucoxanthin had been successfully encapsulated in heteroprotein complex coacervates [21]. The FTIR spectra revealed that phlorotannin had a broad absorption peak around 3410 cm^−1^ due to the O-H stretching vibrations [22]. An absorption band of the carbonyl group from PVP was detected around 1658 cm^−1^ [23]. Phlorotannin was bound to PVP by hydrogen bonds between the hydroxyl group of phlorotannin and the carbonyl group of PVP. The bond was responsible for the cross-linking of the two compounds, facilitating their precipitation in the alcohol solution and the formation of PPNPS [23].

The DSC (differential scanning calorimetry) thermogram of phlorotannin presented an endothermic peak at 130 °C, indicating its melting point, as shown in Figure 1d. PVP is an amorphous polymer and, for this reason, it did not present a clear melting peak. Therefore, the endothermic peak present was attributed to the evaporation of residual moisture. An endothermic peak of phlorotannin could not be seen in the nanoparticles, probably due to the low phlorotannin concentration and its encapsulation by PVP. X-ray diffractometry is a useful way to determine the crystalline formation for the nutrients or drugs encapsulated between nanoparticles [24]. The XRD (X-ray diffractometry) analysis of PPNPS was performed as shown in Figure 1e. Phlorotannin showed major sharp peaks at 2θ diffraction angles 20° and 50°, which confirmed the phlorotannin’s crystalline nature. The XRD pattern of pure PVP did not show crystalline peaks because PVP is an amorphous polymer. Meanwhile, in the physical mixture spectrum, the characteristic phlorotannin peaks with a low level were also found at the same positions as that in phlorotannin powder, indicating that phlorotannin in the mixture is also crystalline. However, crystalline peaks disappeared in the XRD pattern of PPNPS at the ratio of 1:6, 1:8 and 1:10. In fact, this crystallinity modification could be attributed to PVP, which may have surrounded the crystal aggregates of phlorotannin during the preparation of PPNPS, thus forming an amorphous shell [25]. These results are consistent with previous reports which showed that the physical state of lutein was converted from crystalline to amorphous due to encapsulation and the chemical interactions between lutein and PVP during nanoparticle preparation [20]. These characterization results combined with the embedding rate of phlorotannin show that the ratio of 1:8 would be appropriate for making PPNPS based on its good particle size and effective embedding rate.

**Figure 2:**
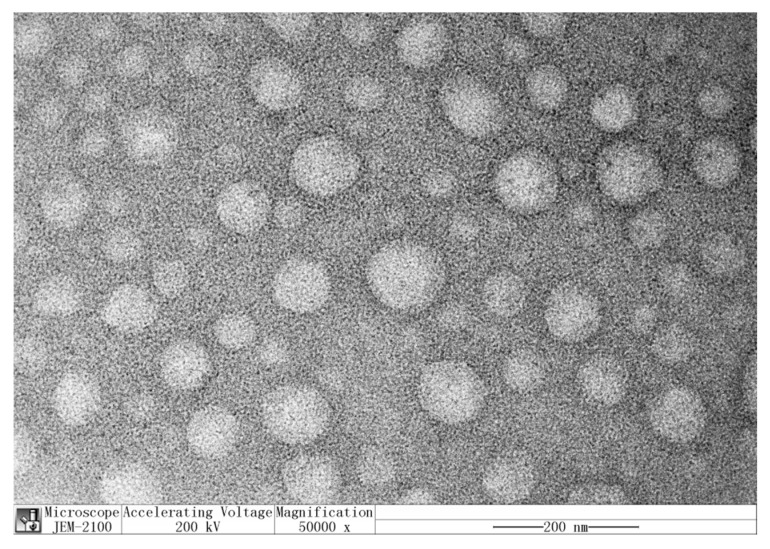
TEM (transmission electron microscopy) images of PPNPS. The concentration of PPNPS was 1 mg/mL.

Figure 2 presents the transmission electron microscopy (TEM) image of PPNPS in a phlorotannin:PVP mass ratio of 1:8 (*w/w*). The TEM image shows highly uniform and monodisperse nanoparticles without agglomeration [26] and which were mainly composed of spherical nanoparticles, similar to the results reported by do Prado Silva, J.T. et al. [23]. Most particles were visible at ~40–50 nm, which was consistent with the particle size results.

### 2.2. Stability of Phlorotannin

**Figure 3:**
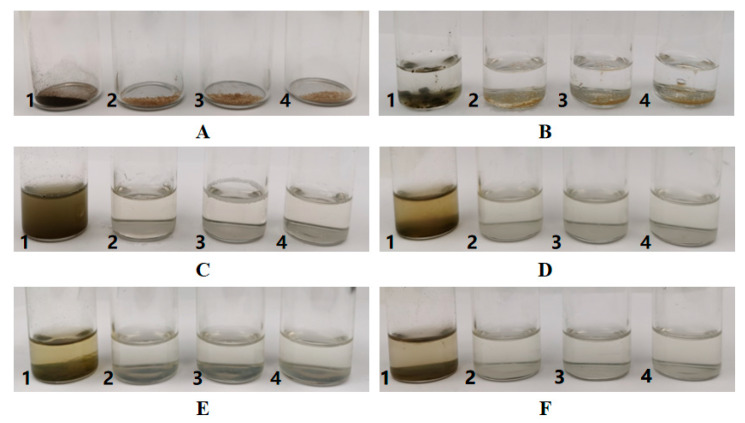
Stability of free phlorotannin (1) and PPNPS (1:6(2), 1:8(3), 1:10(4)) at room temperature (22 ± 3 °C) (A: Original state; B: Adding water; C: Mixture; D: Standing for 5 days; E: Standing for 10 days; F: Standing for 15 days). The concentration of every sample was 20 mg/mL (100 mg solid sample mixed with 5 mL water).

The storage stability of phlorotannin was evaluated under the same conditions over a 15-day duration, as shown in Figure 3. The original state of phlorotannin and PPNPS (1:6, 1:8, 1:10) were incubated in glass tubes with the same amounts. After adding the same amount of water and mixing samples, the PPNPS were dissolved in water. However, phlorotannin did not dissolve in the water and formed a green suspension that was prone to sedimentation as shown in Figure 3. After incubation for 5 days at room temperature (22 ± 3 °C), the PPNPS were stable in water without precipitation. This phenomenon was also found after standing for 10 days and 15 days. These results suggest that the deposition rate of phlorotannin out of the nanoparticles was very low. The PVP-embedded phlorotannin, on the other hand, could be uniformly dissolved in water. For dispersions, no precipitation was observed, indicating the absence of phlorotannin expulsion from PVP. These results indicate that encapsulation effectively improved the stability of phlorotannin. This proved that the deposition rate of nanoencapsulated phlorotannin was lower than that of unembedded ones. It also showed that the stability of the product was quite good. Similar detection methods and results were also reported in the study of astaxanthin-loaded core-shell nanoparticles consisting of chitosan oligosaccharides and poly (lactic-co-glycolic acid) [27].

### 2.3. Controlled Release of Phlorotannin

**Figure 4:**
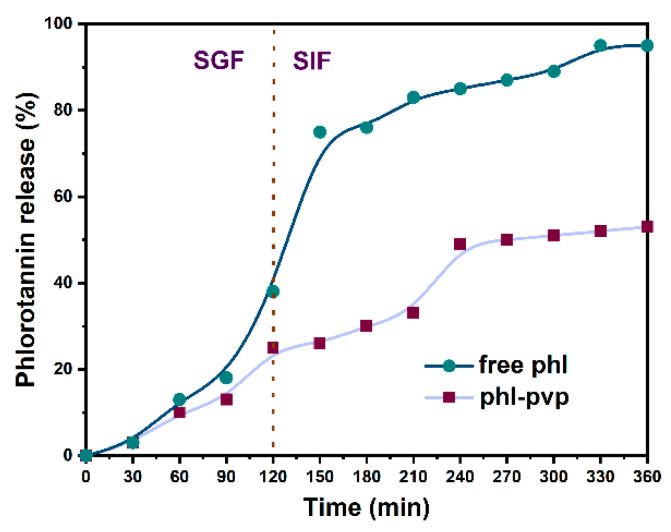
Release profile of free phlorotannin and PPNPS (1:8, *w/w*) in simulated gastrointestinal fluids.

An in vitro release profile is usually used to predict in vivo behavior, drug dosage, release mechanism and kinetics of encapsulated drugs [25]. As shown in Figure 4, the release characteristics of free phlorotannin and phlorotannin from the PPNPS under simulated gastrointestinal conditions were studied. Free phlorotannin showed a 39.68 ± 2.96% burst release within 2 h in a simulated gastric fluid (SGF). Subsequently, it was continuously released in a simulated intestinal fluid (SIF) in the next 4 h and the final cumulative percentage of the released phlorotannin reached 94.72 ± 1.37%. In contrast, under simulated gastrointestinal conditions, phlorotannin in PPNPS showed slower and sustained release kinetics with a total release percentage of approximately 52.76 ± 0.94%. Based on these results, PVP as a shell material could slow down the release rate of phlorotannin. As shown in other investigations, encapsulation with PVP is an approach for enhancing the stability and bioavailability of poorly water soluble drugs [28] and our results were consistent with this research. Other investigations reported the slow release in gastrointestinal conditions of other bioactive compounds such as curcumin [29], thyme essential oil [30], phloretin [19] and fucoxanthin [31], which were also in agreement with our study.

### 2.4. Antioxidant Activity and Cytotoxicity of PPNPS

**Figure 5:**
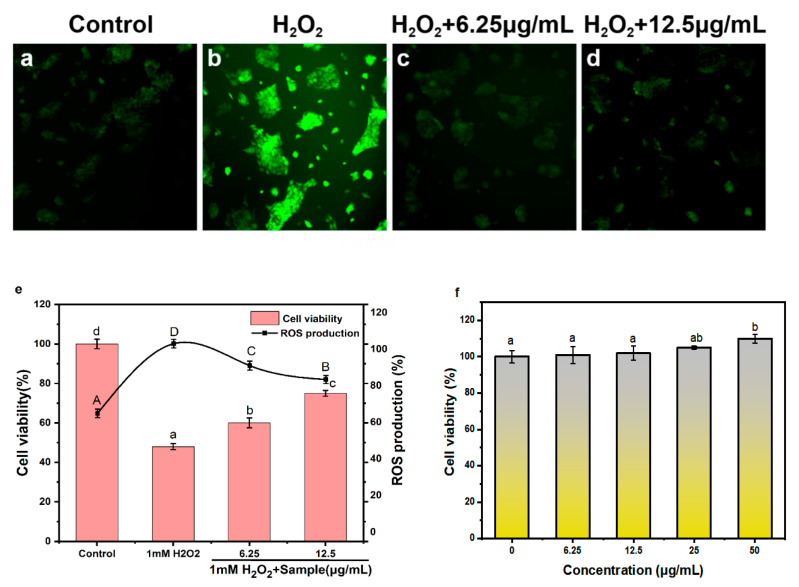
Antioxidant activity and cytotoxicity of HaCaT keratinocytes. (**a**–**d**) Fluorescent images of HaCaT keratinocytes treated with H_2_O_2_ and PPNPS (1:8) and stained with H_2_DCFDA probe. (**e**) Cell viability and relative fluorescence intensity of HaCaT keratinocytes treated with H_2_O_2_ and PPNPS (1:8, *w/w*) at different concentrations. (**f**) Cytotoxicity of HaCaT keratinocytes treated with PPNPS (1:8, *w/w*) at different concentrations. Data are expressed as the mean ± SD. Multiple group comparisons were performed using one-way ANOVA; data that are not denoted with the same letter are significantly different (*p* < 0.05).

Overexposure to reactive oxygen species (ROS) can cause oxidative stress, which can damage essential cell macromolecules (such as proteins, lipids and DNA), thereby increasing risks of several chronic human diseases such as cancer, inflammation and neurodegenerative diseases [32]. Hydrogen peroxide (H_2_O_2_) can induce oxidative damage to cells by increasing the production of ROS. We hypothesized that PPNPS may have protective effects on H_2_O_2_-induced oxidative damage. To test this hypothesis, the production of ROS was measured in HaCaT keratinocytes (Figure 5) upon peroxide treatments. Fluorescent images of HaCaT keratinocytes stained with H_2_DCFDA probes were shown in Figure 5a–d. In the fluorescence images, the H_2_O_2_ treatment group had the strongest fluorescence intensity and after the PPNPS treatment the fluorescence decreased. As expected, H_2_O_2_ treatment significantly increased cellular ROS production in HaCaT keratinocytes, while PPNPS treatment significantly reduced ROS production. When the concentrations of PPNPS treatment were 6.25 and 12.5 μg/mL, the proportion of ROS production decreased by 12 and 18%, respectively. The results are consistent with previous reports that phlorotannin could be developed as a potential ROS inhibitor [33]. In terms of the effect on cell viability, the cell viability of HaCaT keratinocytes treated with 1 mM hydrogen peroxide was 47.90 ± 1.49%, and that of HaCaT keratinocytes treated with 6.25 and 12.5 μg/mL PPNPS significantly increased to 60.02 ± 2.50% and 75.71 ± 1.51% (*p* < 0.05), respectively. That meant that PPNPS could improve the cell viability of oxidized HaCaT keratinocytes and could be potentially used in skin antioxidants. The cytotoxicity of the PPNPS was determined using HaCaT keratinocytes. Four selected concentrations of 6.25, 12.5, 25 and 50 µg/mL PPNPS (1:8, *w/w*) were incubated with HaCaT keratinocytes for 24 h to detect whether PPNPS could produce any toxic effects. As shown in Figure 5f, the cell viabilities of the four concentrations were 100.54 ± 4.69%, 101.72 ± 4.01%, 104.98 ± 0.81%, 109.88 ± 2.42%, respectively. The cell viabilities among 6.25, 12.5 and 25 µg/mL PPNPS (1:8, *w/w*) were not significant (*p* > 0.05), but the cell viability significantly increased at the concentration of 50 µg/mL PPNPS (1:8, *w/w*) (*p* < 0.05). These results indicated that PPNPS were not cytotoxic to HaCaT keratinocytes. It can safely be used on skin or as functional chemicals in cosmetics.

## 3. Materials and Methods

### 3.1. Samples and Chemicals

Phlorotannin (80% purity) from kelp was obtained from Hubei Yongkuo Technology Co., Ltd. Ethanol (99.5%, Neon) and polyvinylpyrrolidone (40.000 g·mol^−1^, Sigma-Aldrich) were used in the extraction and encapsulation of the phlorotannin. HaCaT keratinocytes came from Shanghai Tongpai Technology Co., Ltd. and were cultured in Dulbecco’s Minimum Essential Medium (DMEM), containing 100 U/mL penicillin, 100 U/mL streptomycin and 10% FBS. DMEM and trypsin (0.25% with EDTA) were purchased from Gibco (Thermo Fisher, Fair Lawn, NJ, USA). Penicillin, streptomycin solution and PBS were obtained from HyClone (Logan, UT, USA). Fetal bovine serum (FBS) was obtained from Sangon Biotech Co., Ltd. (Shanghai, China). Dimethyl sulfoxide (DMSO) was obtained from Yeasen Biotech Co., Ltd. (Shanghai, China). MTT and 2′,7′-dichlorodihydrofluorescein diacetate (H_2_DCFDA) were obtained from Baoxidi Co., Ltd. (Beijing, China). Pepsin and pancreatin were purchased from Sigma. Other reagents were obtained from Sangon Biotech Co., Ltd. (Shanghai, China).

### 3.2. Preparation of PPNPS

Initially, PVP was dissolved in ethanol under magnetic stirring until translucent solutions were obtained. Phlorotannin extracts with 80% purity were washed with water until colorless and were centrifuged to discard the supernatant to remove highly polar substances. They were then washed with CHCl_3_ until colorless to remove weakly polar substances and the obtained purified phlorotannin was dissolved in 100% ethanol. The purified phlorotannins with purity of 90.24 ± 4.47% based on Folin–Ciocalteu method were to be used for the next steps. The same weight amounts of phlorotannin in ethanol solution were added in different proportions of PVP translucent solutions which were initially prepared as the ratio of 1:2, 1:4, 1:6, 1:8, 1:10, 1:12, 1:14 (phlorotannin:PVP, *w/w*) were added to phlorotannin solution, and the mixtures were stirred evenly. The obtained mixtures were sonicated for 15 min under pulse condition of 30 s on and 10 s off (Sonics Vibra-Cell™, and 1/8′tip). Finally, the solvent was evaporated in a circulation oven at 30 °C for 24 h, then the samples were freeze-dried in lyophilizer (Scientz-10ND, ZheJiang, China). This nanoparticle-formed mechanism could be due to the hydrogen bond formation between PVP and phlorotannin inside the nanoparticles.

### 3.3. Nanoparticle Characterization

Dispersion Technology (DT Zeta & Size 1202, Dispersion Technology, Inc. Bedford Hills, NY USA) was used to measure the particle size distribution and mean particle size of the PPNPS. The thermal properties of PPNPS were determined by DSC (DSC250, TA Instrument, New Castle, DE, USA). Nanoparticles, pure compounds (phlorotannin and PVP) and their physical mixture (a phlorotannin:PVP ratio of 1:1 *w/w* manually mixed) were weighed (5 to 10 mg) in aluminum pans. Samples were heated from 20 to 300 °C at 20 °C min^−1^ under a nitrogen flow of 50 mL·min^−1^. FTIR spectra analysis (PerkinElmer, Norwalk, CT, USA) was performed from 4000 to 450 cm^−1^, using a KBr disk with 1% finely ground samples in order to identify chemical interactions between phlorotannin and PVP. X-ray powder diffraction patterns of phlorotannin, PVP, physical mixture of phlorotannin and PVP, PPNPS (1:6), PPNPS (1:8) and PPNPS (1:10) were taken at ambient temperature and were obtained with an X-ray diffractometer (XRD7000, JEOL, Tokyo, Japan). The amount of phlorotannin in all samples was the same. The following conditions were used: X-ray tube target of Cu, voltage of 40.0 (kV), current of 30.0 (mA), divergence slit of 1.0 (deg), scatter slit of 1.0 (deg), receiving slit of 0.3 (mm), drive axis of Theta-2, scan range of 10.0–70.0, scan mode of Continuous Scan, scan speed of 5.0 (deg/min), sampling pitch of 0.02 (deg) and preset time of 0.24 (sec). Morphological characterization of the nanoparticles was performed using TEM (JEM 2100, Shimadzu Corporation, Japan). Diluted samples were dripped onto 300 mesh parlodium-covered copper grids and were stained with 2% uranium acetate. Grids were dried at room temperature and then analyzed.

### 3.4. Determination of Stability

Phlorotannin and PPNPS with different ratios of 1:6, 1:8, 1:10 with added water were kept at room temperature 22 ± 3 °C without light irradiation for 15 days to observe the storage stability.

### 3.5. Determination of Controlled Release In Vitro

In vitro release experiments of the encapsulated phlorotannin were carried out under two simulated digestive fluids. According to Oliyaei’s methods [31] with minor modifications, 0.1 M HCl solution of pH 1.2 with pepsin (0.3% *w*/*v*) was used as a SGF and 0.1 M NaHCO_3_ solution of pH 7.5 with pancreatin (0.1% *w*/*v*) was usedas a SIF. Approximately 0.5 g of PPNPS (1:8) was added to 10 mL SGF and vortexed in the dark for 120 min. The system was maintained at 37 °C under shaking, then immersed in 10 mL of SIF and incubated under the same condition for 240 min. A sample was taken every 30 min. The release of phlorotannin was determined using high-performance liquid chromatography (HPLC, Shimadzu SPD 20A, Japan). Methanol was used as the mobile phase at 0.8 mL·min^−1^, in a C_18_ column (4.6 × 250 mm and particle size of 5 µm) at 40 °C. The elution gradient went from 100 to 85% methanol in 10 min. Detection was performed by a diode array detector (DAD) at 254 nm. The release of the phlorotannin was calculated according to Equation (1):

Fractional release (%) = (1 − M_t_/M_0_) × 100 (1)

where M_0_ is the amount of phlorotannin initially encapsulated and M_t_ is the amount of phlorotannin remaining in the carriers at a given incubation time.

### 3.6. Cell Culture

HaCaT keratinocytes were thoroughly mixed in DMEM medium supplemented with 10% fetal bovine serum, 100 U/mL penicillin and 100 U/mL streptomycin, and cultured in a humidified atmosphere of 5% CO_2_ at 37 °C according to the instructions from Shanghai Tongpai Technology Co., Ltd. When the cells reached a certain degree of confluence, they were washed with PBS for dead cells, digested with trypsin-EDTA, diluted to a suspension in fresh medium and set aside.

### 3.7. Antioxidant Activity Analysis

A total of 100 μL of HaCaT keratinocytes (1 × 10^5^/mL) was seeded onto a 96-well microtiter plate. After incubation in 5% CO_2_ incubator at 37 °C for 24 h, the cells were treated with 1mM hydrogen peroxide (H_2_O_2_) for 1 h or without treatment (control). Then the cells were treated with varying concentrations of PPNPS (1:8) for another 23 h in 5% CO_2_ incubator at 37 °C. Then cells were incubated with MTT (5 mg/mL) for 4 h to stain the cells. The MTT solution was then removed and 150 μL dimethyl sulfoxide (DMSO) was added and shaken well. The absorbance at 570 nm was determined by a microplate reader (Tecan Infinite, Männedorf, Switzerland). The control group refers to the cells without any treatment. The results were calculated and expressed as the cell viability according to Equation (2):

Cell viability (%) = (A_sample_ /A_control_) × 100 (2)

### 3.8. Intracellular ROS Detection

The generation of reactive oxygen species (ROS) was measured according to previous reports [34,35] with slight modifications. ROS can be evaluated by testing changes in fluorescence intensity of H_2_DCFDA, which is the most commonly applied method for quantitative examination of intracellular ROS. The intensity of green fluorescence is proportional to the level of ROS. HaCaT keratinocytes with a density of 1.0 × 10^5^ cells/mL were seeded in a 12-well plate (2 mL/well) for 24 h at 37 °C in a humidified incubator containing 5% CO_2_. Then, cells were treated with 1 mM H_2_O_2_ for 1 h then the cells were dealt with varying concentrations of PPNPS (1:8) for another 1 h in 5% CO_2_ incubator at 37 °C. The control group meant the cells without any treatment. Finally, treated cells were incubated with H_2_DCFDA for 30 min in PBS in 5% CO_2_ incubator at 37 °C. The fluorescence intensity at varying circumstances was tracked by a fluorescent inverted microscope (Olympus IX73, Kyoto, Japan).

HaCaT keratinocytes with a density of 1.0 × 10^5^ cells/mL were seeded in a 12-well plate (2 mL/well) for 24 h at 37 °C in a humidified incubator containing 5% CO_2_. Then, cells were treated with 1 mM H_2_O_2_ for 1 h then the cells were treated with varying concentrations of PPNPS (1:8) for another 1 h in 5% CO_2_ incubator at 37 °C. The control group meant the cells without any treatment. Finally, treated cells were incubated with H_2_DCFDA for 30 min in PBS in 5% CO_2_ incubator at 37 °C. After these treatments, cells were washed in PBS, trypsinized and collected by centrifugation. Then the cells were washed twice more before fixing with PBS, and were evenly distributed in a 96-well fluorescent plate. ROS production values were determined by a fluorescence microplate reader (Tecan Infinite 200 pro, Switzerland); the excitation wavelength was 485 nm and the emission wavelength was 535 nm. ROS production was calculated according to Equation (3):

ROS production (%) = (A_sample group_/A_H2O2 group_) × 100 (3)

### 3.9. In Vitro Cytotoxicity

The MTT assay was used to measure cell metabolic activity, cytotoxicity, proliferation and viability. Cell viability was determined by measuring the amount of tetrazolium salt in the viable cells of each sample using untreated cells as controls [36]. A cell culture model was used to determine the relative cytotoxicity of the nanoparticles by cell viability according to Liu et al.’s methods [37]. In a nutshell, 100 μL of HaCaT keratinocytes (1 × 10^5^/mL) was seeded onto a 96-well microtiter plate at 37 °C in a humidified atmosphere with 5% CO_2_ for 24 h. The cells were treated with varying concentrations of PPNPS for another 24 h. The control group meant the cells without any treatment. Then cells were incubated with MTT (5 mg/mL) for 4 h to stain the cells. The MTT solution was then removed and 150 μL dimethyl sulfoxide (DMSO) was added and shaken well. The absorbance at 570 nm was determined by a microplate reader (Tecan Infinite, Switzerland). The results were calculated and expressed as the cell viability according to Equation (4):

Cell viability (%) = (A_sample_/A_control_) × 100 (4)

### 3.10. Statistical Analysis

Each experiment was repeated at least 3 times and analyzed for variance. The results were expressed as the means ± standard deviation. All data were performed with a one-way analysis of variance (ANOVA) and analyzed by SPSS 16.0 (SPSS Inc., 2001, Chicago, IL, USA) where *p* < 0.05 was considered as the significance level.

## 4. Conclusions

In this study, phlorotannin was encapsulated in PVP shells to form PPNPS with relatively small, smooth spheres which were analyzed by TEM. The physicochemical characteristics which were analyzed by FTIR, DSC, X-ray and embedding rate proved that the best ratio of phlorotannin to PVP was 1:8 when phlorotannin was effectively encapsulated. DSC and X-ray analysis also showed that the phlorotannin was encapsulated inside the nanoparticles in an amorphous state, which should enhance its solubility and bioavailability. A sustained kinetic release profile was observed for the PPNPS in simulated GI conditions. Moreover, the PPNPS exhibited an outstanding antioxidant activity on HaCaT keratinocytes. In conclusion, our study demonstrated that PPNPS has the potential to be used as an effective oral delivery vehicle as well as in cosmetics to protect oxidative damage to the skin. The present work established a baseline for further investigation. In the next step, we will conduct an in vivo study to evaluate antioxidant activities of the nanoencapsulation.

## Abbreviations

PPNPS	phlorotannin@pvp nanoparticles
ROS	reactive oxygen species
PVP	polyvinylpyrrolidone
TEM	transmission electron microscopy
FTIR	fourier transform infrared
DMEM	Dulbecco’s Minimum Essential Medium
FBS	fetal bovine serum
DMSO	dimethyl sulfoxide
H_2_DCFDA	2′,7′-dichlorodihydrofluorescein diacetate
DSC	differential scanning calorimetry
XRD	X-ray diffraction analysis
HPLC	high-performance liquid chromatography
